# The effect of trisomic chromosomes on spatial genome organization and global transcription in embryonic stem cells

**DOI:** 10.1111/cpr.13639

**Published:** 2024-03-29

**Authors:** Mengfan Li, Junsheng Yang, Rong Xiao, Yunjie Liu, Jiaqi Hu, Tingting Li, Pengze Wu, Meili Zhang, Yue Huang, Yujie Sun, Cheng Li

**Affiliations:** ^1^ Center for Bioinformatics, School of Life Sciences, Center for Statistical Science, Peking‐Tsinghua Center for Life Sciences Peking University Beijing China; ^2^ State Key Laboratory of Membrane Biology, School of Life Sciences, and Biomedical Pioneering Innovation Center (BIOPIC) Peking University Beijing China; ^3^ State Key Laboratory of Common Mechanism Research for Major Diseases, Department of Medical Genetics Institute of Basic Medical Sciences, Chinese Academy of Medical Sciences, School of Basic Medicine, Peking Union Medical College Beijing China; ^4^ School of Health Humanities Peking University Beijing China; ^5^ State Key Laboratory of Proteomics Institute of Basic Medical Sciences, National Center of Biomedical Analysis Beijing China

## Abstract

Aneuploidy frequently occurs in cancer and developmental diseases such as Down syndrome, with its functional consequences implicated in dosage effects on gene expression and global perturbation of stress response and cell proliferation pathways. However, how aneuploidy affects spatial genome organization remains less understood. In this study, we addressed this question by utilizing the previously established isogenic wild‐type (WT) and trisomic mouse embryonic stem cells (mESCs). We employed a combination of Hi‐C, RNA‐seq, chromosome painting and nascent RNA imaging technologies to compare the spatial genome structures and gene transcription among these cells. We found that trisomy has little effect on spatial genome organization at the level of A/B compartment or topologically associating domain (TAD). Inter‐chromosomal interactions are associated with chromosome regions with high gene density, active histone modifications and high transcription levels, which are confirmed by imaging. Imaging also revealed contracted chromosome volume and weakened transcriptional activity for trisomic chromosomes, suggesting potential implications for the transcriptional output of these chromosomes. Our data resources and findings may contribute to a better understanding of the consequences of aneuploidy from the angle of spatial genome organization.

## Graphical abstract

Chromosome territories, inter‐chromosomal interactions and gene transcription in wild‐type (WT) and trisomic mouse embryonic stem cells (mESCs). Hi‐C, RNA‐seq and imaging data showed reduced chromosome volume of both trisomic and disomic chromosomes. Radial location of chromosomes and inter‐chromosomal interaction preferences were retained in trisomic mESCs, and chromosome intermingling regions were associated with active transcription in both WT and trisomic mESCs. Using coupled chromosome painting and nascent RNA imaging in single cells, we observed contracted chromosome volume concomitant with weakened transcriptional activity for chromosome 8 in trisomy 8 (Ts8) mESC compared with WT mESC, but no significant radial relocation of chromosome territories in Ts8 mESC.

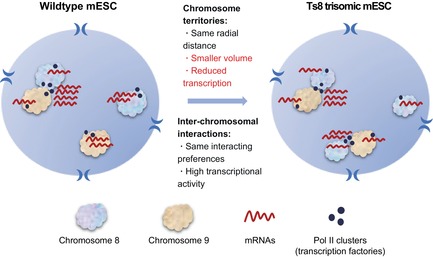

## 1 INTRODUCTION

The diploid genome in humans and other mammalian species generates individual diversity through sexual reproduction and serves as a buffer against the deleterious effects of mutations in a single allele.^1^ However, cell division errors can lead to aneuploidy in daughter cells, characterized by the gain or loss of partial or whole chromosomes.^2^ A specific form of aneuploidy is trisomy, defined as the existence of three copies of a particular chromosome in a diploid cell. Trisomy recurrently occurs in cancers, birth defects and cultured pluripotent stem cells,^3^, ^4^, ^5^ and is associated with diverse phenotypes such as increased susceptibility to leukaemia and impaired cognition in individuals with Down syndrome.^6^

The physiological effect of aneuploidy is partly explained by copy number alterations associated with aneuploidy, which have a dosage effect on mRNA and protein expression levels.^7^, ^8^, ^9^, ^10^ Aneuploidy can impair homeostasis of protein complexes, leading to promiscuous protein interactions and sensing of aneuploidy by the p53 pathway.^11^, ^12^, ^13^, ^14^ Cumulative dosage effects of multiple genes have oncogenic or tumour‐suppressing functions.^15^, ^16^, ^17^ Such transcriptional dosage effect of aneuploidy may mediate the altered growth fitness and sensitivity to protein folding inhibitors in aneuploidy models of yeast and mouse cells,^18^, ^19^, ^20^ providing a rationale to explain aneuploidy's selective advantages during disease progression and to explore its vulnerabilities for treatments.^21^, ^22^ However, whether and how aneuploidy affects the epigenome, such as DNA methylation and spatial chromatin organization, is less understood.^23^, ^24^

Recently, the 3D genome structures are increasingly studied by sequencing and imaging techniques.^25^, ^26^ These techniques have discovered hierarchical chromatin structures such as chromosomal territories (CT), A/B compartments, topologically associating domains (TADs) and chromatin loops.^27^, ^28^ Importantly, the dynamics of 3D genome is associated with gene transcription during various biological and pathological processes.^29^, ^30^, ^31^, ^32^, ^33^ These findings have motivated studies on the relationship between 3D genome and aneuploidy, both of which could affect gene transcription. In cultured human embryonic cells with trisomies 18 or 21, trisomic chromosomes have lower CT volumes but remain at similar nuclear positions as their counterparts in normal cells.^34^ A recent study observes that trisomy 21 disrupts genome organization and transcription to promote senescent phenotype in neural progenitors.^35^ In cancer cells, chromatin compartments, TADs, and intra‐ and inter‐chromosomal interactions have been found to be altered relative to normal cells.^36^, ^37^, ^38^, ^39^, ^40^, ^41^ However, the cancer samples used in these studies harbour various mutations, structural variations, in addition to aneuploidy, preventing the study of the specific effect of aneuploidy on genome organization and gene expression.

To overcome these limitations, in this study, we compared wild‐type (WT) mouse embryonic stem cells (mESCs) with four different WT mESC‐derived trisomic mESC lines, each carrying an extra chromosome. Employing a comprehensive approach integrating Hi‐C, RNA‐seq and advanced imaging techniques, we explored the specific effects of trisomic chromosomes on spatial chromatin organization and gene expression while minimizing confounding factors. Our results supported that trisomy in mESCs does not significantly affect intra‐ and inter‐chromosomal interactions. Instead, the predominant effects on gene expression stem from the gene dosage effect and global transcriptional regulation. We also observed a reduction in the volume of trisomic chromosomes, concurrent with the moderate dosage compensation for these chromosomes.

## 2 RESULTS

### 2.1 Compartments and TADs are unaffected by trisomic chromosomes

Previous studies show that hyperdiploid leukaemia cells have reduced TAD boundaries and strength associated with gene expression dysregulation,^42^ and the gain of chromosome 7 in a colon cell line results in global alteration of gene expression and chromatin structures involving both the trisomic and other chromosomes.^43^ To minimize confounding genome mutations in cancer samples, we employed WT and trisomic mESCs to investigate the relationship between aneuploidy and genome organization. We have previously generated isogenic mESC lines that carry an extra copy of single chromosomes to study the effect of aneuploidy on cell proliferation and tumorigenesis. These trisomic mESCs show increased neoplastic potential and reduced differentiation potential compared with WT mESC.^44^

To study the effect of trisomy on gene transcription and genome organization, we conducted RNA‐seq and Hi‐C experiments for both WT and trisomic mESCs harbouring an extra copy of chromosome 6, 8, 11 and 15, respectively (named as Ts6, Ts8, Ts11 and Ts15). We performed in situ Hi‐C experiments for WT and trisomic mESCs following the standard protocol.^28^ The data exhibited typical statistics for reads filtering and *cis*/*trans* chromatin interaction proportions in Hi‐C experiments (Figure S1A, Supplementary File 1), with high reproducibility observed between replicate samples (Figure S1B). In addition, our Hi‐C interaction matrices were highly similar to a public Hi‐C data set of mESCs (E14 cell line), which was derived from the same 129 inbred mouse strain as our mESCs (Figure S1C,D).

**FIGURE 1:**
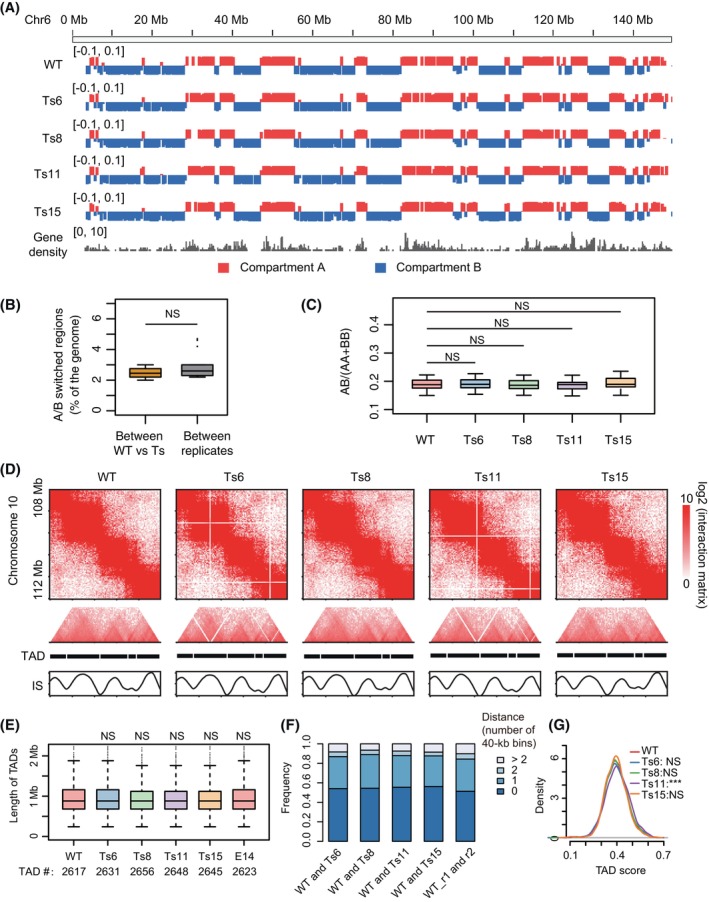
Compartments and TADs are unaffected by trisomic chromosomes. (A) Representative genomic tracks showing A/B compartments in WT and trisomic mESCs. (B) The proportions of genomic regions with switched A/B compartment status between WT and trisomic mESCs (left box) and between replicates of each cell line (right box). Two‐sided Wilcoxon rank‐sum test was used. *p* Value = 0.4252. NS: not significant. (C) The distribution of A/B compartment segregation scores in WT and trisomic mESCs. The segregation score of a chromosome is the ratio of the interactions of different types of compartments (A–B interaction) to the sum of the interactions of the same type of compartments (A–A interaction and B–B interaction). Each boxplot consists of the segregation scores of all individual chromosomes. Two‐sided Wilcoxon rank‐sum test was used. From left to right, *p* value = 0.7788, 0.6588, 0.6205 and 0.4945. NS: not significant. Representative chromatin interaction heatmaps, TAD domains and insulation scores (IS) in different mESC samples (bin resolution: 40 kb). (E) The distribution of TAD lengths and the number of TADs in different mESC samples and the E14 cell line. Two‐sided Wilcoxon rank‐sum test was used to compare each trisomic cell and E14 with WT, respectively. From left to right, *p* value = 0.54, 0.3093, 0.3164, 0.689 and 0.6493. NS: not significant. (F) The TAD boundaries are largely conserved (distance <1 bin) between WT and trisomic mESCs. More than 50% of the TAD boundaries in WT and trisomic mESCs were overlapped, and about 90% of the TAD boundaries were shifted within 80 kb. (G) The distributions of TAD compaction scores in WT and trisomic mESCs are overlapping. Two‐sided Wilcoxon rank‐sum test was used to compare each trisomic cell with WT, respectively. From Ts6, Ts8, Ts11 to Ts15, *p* value = 0.7785, 0.2301, 1.776e−06 and 0.642. ***p Value < 0.001, NS: not significant. mESC, mouse embryonic stem cell; WT, wild‐type.

To compare chromatin structures between WT and trisomic mESCs, we first used the ICE algorithm to correct for copy number effects on Hi‐C interactions (Figure S2A,B).^45^, ^46^ The Hi‐C interaction matrices and A/B compartments of WT and trisomic mESCs were overall highly similar (Figures 1A and S1E), with the proportion of A/B compartment switched regions similar when comparing between WT and trisomic mESCs or between replicate samples (Figure 1B). The A/B segregation scores were not significantly different between WT and trisomic mESCs (Figure 1C), confirming the maintained A/B compartmentalization in the trisomic mESCs. We next identified TAD boundaries as local minimums of insulation scores (Figure 1D).^47^ The lengths of TADs and the location of TAD boundaries were highly concordant between WT and trisomic mESCs (Figure 1E,F). Furthermore, TAD compactness score, defined as the proportion of chromatin interaction counts within a TAD among all interactions involving the TAD, was distributed similarly between WT and trisomic mESCs (Figure 1G). These results indicate that the presence of an extra chromosome in trisomic mESCs does not affect intra‐chromosomal chromatin structures at the compartment or TAD level.

### 2.2 Inter‐chromosomal interactions are non‐random and preserved in trisomic mESCs

**FIGURE 2:**
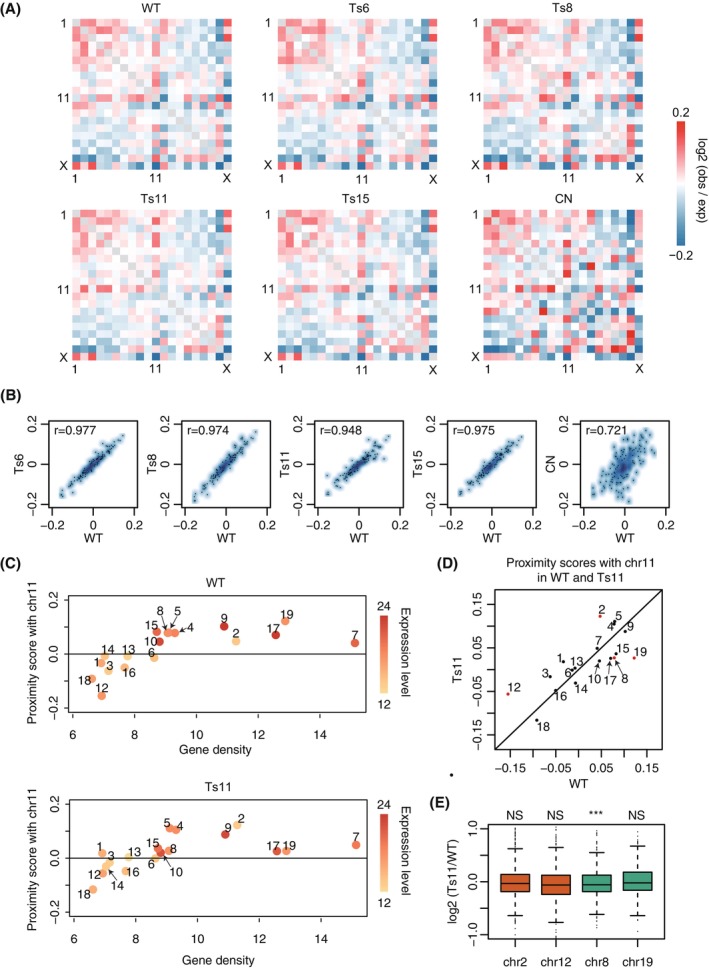
Inter‐chromosomal interactions are non‐random and preserved in trisomic mESCs. (A) Genome‐wide inter‐chromosomal proximity score matrices of the mESCs and mouse cortical neurons (CNs). (B) Scatter plots of the inter‐chromosomal proximity scores of sample pairs. Pearson's correlation coefficients are displayed in the figures. (C) The relationship between the inter‐chromosomal proximity score of each chromosome with chromosome 11 (*y*‐axis) and the gene density (gene number/Mb) of each chromosome (*x*‐axis). The chromosomes are coloured by chromosome‐wide expression level, defined as the average FPKM (fragments per kilobase per million mapped reads) of all genes on each chromosome. Top: WT mESC; bottom: Ts11 mESC. (D) Scatter plot of inter‐chromosomal proximity scores between the indicated chromosomes with chromosome 11 in WT (*x*‐axis) and Ts11 (*y*‐axis) mESCs. Chromosomes whose scores change most between the two samples are labelled in red. (E) The distribution of gene expression changes between WT and Ts11 mESCs for genes on chromosomes 2 and 12 (increased proximity score with chromosome 11 in Ts11), and on chromosomes 8 and 19 (decreased proximity score with chromosome 11 in Ts11). One‐sample one‐sided Wilcoxon signed‐rank tests. H0: the median is greater than 0 (for chromosomes 2 and 12); the median is less than 0 (for chromosomes 8 and 19). From left to right, *p* value = 0.9969, 1, 8.943e−5 and 0.4255. ***p value < 0.001, NS: not significant. mESC, mouse embryonic stem cell; WT, wild‐type.

In addition to intra‐chromosomal chromatin interactions, different chromosomes also interact to organize genome structures and regulate gene expression.^48^, ^49^ Such inter‐chromosomal interactions may promote specific chromosomal translocations^50^, ^51^ or change in diseased conditions.^36^, ^52^, ^53^ We therefore examined inter‐chromosomal interactions in WT and trisomic mESCs and their potential relationship with gene transcription. As expected, a trisomic chromosome resulted in increased inter‐chromosomal interactions between the trisomic chromosome and other chromosomes due to copy number effect (Figure S2A,C). To quantify inter‐chromosomal interactions, we calculated an inter‐chromosomal proximity score as the log_2_ ratio of observed to expect interaction counts between pairwise chromosomes (Section 4). Higher proximity scores correspond to more frequent co‐localization of two chromosomes in the nucleus. The proximity score matrices of all chromosomal pairs were highly concordant between WT and trisomic mESCs (Figures 2A,B and S2D), but distinct between mESCs and mouse cortical neurons (CNs) (Figure 2A,B). These results indicate that inter‐chromosomal interactions are non‐random and cell‐type specific, and are preserved in mESCs despite the presence of an extra chromosome.

We next explored potential determinants of non‐random inter‐chromosomal interactions. It is known that the radial position of chromosomes in the nucleus is related to their gene density. Chromosomes with higher and lower gene density tend to be located at the nuclear interior and nuclear periphery, respectively.^54^, ^55^, ^56^ Interestingly, we observed that chromosome 11, the most gene‐rich chromosome in the mouse genome, has strong chromatin interactions with many other chromosomes in all of the mESC samples (Figures 2A and S2D). This prompted us to explore the relationship between inter‐chromosomal interactions and gene density. In fact, other gene‐rich chromosomes tended to be more interactive with chromosome 11 and more actively transcribed than gene‐poor chromosomes (Figures 2C and S3). Such inter‐chromosomal interaction tendency with chromosome 11 was largely maintained in the trisomic mESCs and CNs (Figures 2C,D and S3), and almost did not affect chromosome‐wide gene expression of disomic chromosomes (Figure 2E). Taken together, inter‐chromosomal chromatin interactions are non‐random and more frequent between gene‐rich chromosome pairs, and are unaffected by trisomy in mESCs.

### 2.3 Inter‐chromosomal interactions are associated with active transcription

**FIGURE 3:**
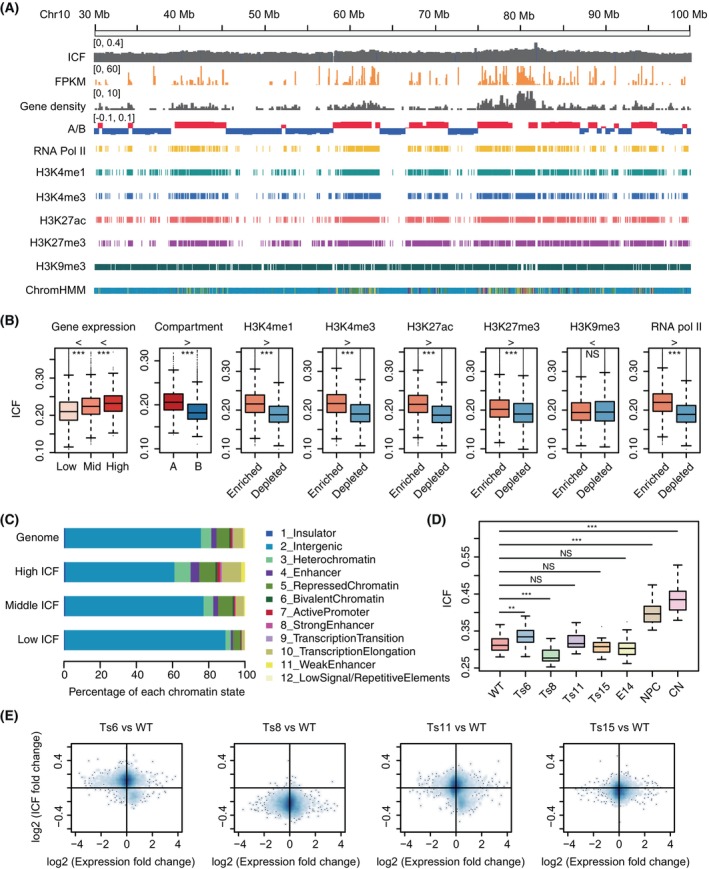
Inter‐chromosomal interactions are associated with active transcription. (A) Representative genomic tracks of WT mESC, showing inter‐chromosomal interaction fraction (ICF), gene expression (FPKM), gene density (gene number/Mb), A/B compartments (red: A, blue: B), and signal peaks of RNA polymerase II occupation, indicated histone modifications, and ChromHMM states (colour legend in panel C). (B) The distribution of ICF values for different chromosomal regions classified by indicated properties. Genes are divided into three groups according to their expression level: low (FPKM <10), middle (10< FPKM <100) and high (FPKM >100). The “>” or “<” signs above the plots indicate the relationship between the medians of two adjacent boxplots. Two‐sided Wilcoxon rank‐sum test was used. From left to right, *p* value <2.2e−16, =9.307e−9, <2.2e−16, <2.2e−16, <2.2e−16, <2.2e−16, <2.2e−16, =0.5209, <2.2e−16. ***p value < 0.001, NS: not significant. (C) The proportion of ChromHMM states in the whole genome or in the chromosomal regions with high, middle or low ICF values. (D) The distribution of ICF values in different samples. Two‐sided *t* test was used. From left to right, *p* value = 0.00575, 1.732e−5, 0.3022, 0.3425, 0.2472, 1.312e−11 and 5.522e−14. **p Value < 0.01, ***p value < 0.001, NS: not significant. (E) Scatter plot of ICF fold changes (*y*‐axis) and gene expression fold changes (*x*‐axis) between WT and trisomic mESCs. mESC, mouse embryonic stem cell; WT, wild‐type.

Inter‐chromosomal interactions have been found to involve epigenetically similar regions such as actively transcribed regions or repressive regions.^48^, ^57^, ^58^ We therefore asked how inter‐chromosomal interactions are related to local gene transcription. We first calculated inter‐chromosomal interaction fraction (ICF) for local chromosomal regions, which is the ratio of inter‐chromosomal interactions to the sum of inter‐ and intra‐chromosomal interactions for a specific region, so that higher ICF corresponds to more frequent interactions between this region and other chromosomes. Chromosomal regions with higher ICF tended to have higher gene density and active transcriptional state, and were located in A compartments (Figure 3A,B). Specifically, RNA polymerase II binding and active histone modifications, such as H3K4me1, H3K4me3, and H3K27ac, were enriched at regions with higher ICF (Figure 3B). Interestingly, the repressive histone modification H3K27me3 was also enriched in regions with higher ICF (Figure 3B). ChromHMM analysis^59^ confirmed that high‐ICF regions were enriched with both active and inactive histone modifications and depleted in intergenic regions (Figure 3C). These results are consistent with the fact that transcriptionally active and repressive protein complexes such as transcriptional factories and Polycomb bodies jointly organize long‐range chromatin interactions.^48^, ^60^

Next, we asked whether trisomic chromosomes perturb inter‐chromosomal interactions of specific chromosomal regions. The distributions of regional ICF values varied across WT and trisomic mESCs (Figure 3D), but were overall lower than that of mouse neural progenitor cells (NPCs) and CNs,^61^ consistent with differentiated cells having increased chromosomal intermingling.^62^ Nevertheless, the changes of ICF values between WT and trisomic mESCs were not correlated with gene expression changes (Figure 3E). These results suggest that inter‐chromosomal interactions are associated with active transcription and are cell‐type specific.

### 2.4 Coupled chromosome painting and nascent RNA imaging validate higher transcriptional activity at chromosome intermingling regions

**FIGURE 4:**
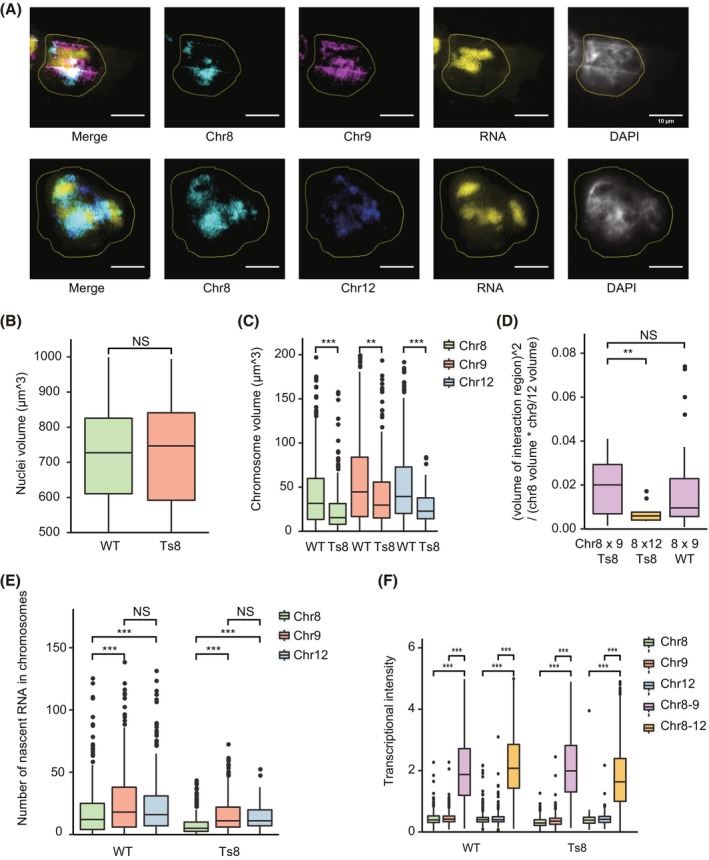
Coupled chromosome painting and nascent RNA imaging reveal higher transcriptional activity at chromosome intermingling regions. (A) Representative simultaneous chromosome painting and nascent RNA imaging results of WT mESCs. (B) The distribution of nucleus volume of WT and Ts8 mESCs. Two‐sided Wilcoxon rank‐sum test, *p* value = 0.95. NS: not significant. From left to right: sample size *n* = 514, 324 nuclei. (C) The distribution of chromosome volume of chromosomes 8, 9 and 12 in WT and Ts8 mESCs. Two‐sided Wilcoxon rank‐sum tests. From left to right: *p* value = 1.7e−11, 0.0023, 2.3e−7; *n* = 350, 315, 424, 210, 354 and 105 chromosomes. **p Value < 0.01, ***p value < 0.001. (D) The distribution of intermingling index between chromosomes pairs in WT or Ts8 mESCs. The intermingling index between the two chromosomes A and B is defined as (volume of interaction region)^2^/(chromosome A volume*chromosome B volume). Two‐sided Wilcoxon rank‐sum tests. From left to right: *p* value = 0.006 and 0.71; *n* = 19, 12 and 42 chromosome pairs. **p value < 0.01, NS: not significant. (E) The distribution of the number of nascent RNAs overlapping with chromosome 8, 9 or 12 in WT and Ts8 mESCs. Two‐sided Wilcoxon rank‐sum tests. From left to right: *p* value = 1.6e−5, 0.00075, 0.23, 1.7e−14, 3.7e−11 and 0.94; *n* = 351, 431, 358, 310, 205 and 100 chromosomes. ***p Value < 0.001, NS: not significant. (F) The distribution of transcriptional intensity of individual chromosomes (8, 9 and 12) or chromosome intermingling regions in WT and Ts8 mESCs. “Chr8–9” (chr8–12) indicates the intermingling region of chromosome 8 and chromosome 9 (chromosome 8 and chromosome 12). Transcriptional intensity is defined as the number of nascent RNAs overlapping a chromosome/region, divided by the volume of the chromosome/region. From left to right: *n* = 325, 399, 1120, 352, 344, 2105, 277, 208, 375, 165, 107 and 741 chromosomes/regions. Two‐sided Wilcoxon rank‐sum tests, all the *p* values < 2.22e−16. ***p Value < 0.001. mESC, mouse embryonic stem cell; WT, wild‐type.

Interphase chromosomes occupy preferred relative nuclear positions and compartmentalized into distinct chromosome territories.^63^, ^64^ It has been observed that in human trisomy 21 cells, certain chromosomal territories shift their preferred radial positions in the nucleus or reduce their volume.^65^ To explore whether trisomic chromosomes affect chromosome territories in mESCs, we used FISH‐based chromosome painting to quantify the volume and radial location of chromosomes 8, 9 and 12 (Figure 4A).^66^, ^67^ Although the volume of the nucleus was comparable between WT and Ts8 mESCs (Figure 4B), the presence of the extra chromosome 8 led to a reduction in chromosome volume, affecting not only the trisomic chromosome 8 but also disomic chromosomes 9 and 12 (Figure 4C). This compaction of chromosome volumes was not due to the relocation of chromosome territories, as the radial location of these chromosomes within the nucleus remained similar between WT and Ts8 mESCs (Figure S4). We next visualized inter‐chromosomal intermingling by using two fluorescence dyes to simultaneously paint two different chromosomes in the same cell.^66^ The intermingling index, defined to characterize the degree of intermingling between chromosomes (Section 4), was significantly higher between chromosomes 8 and 9 than between chromosomes 8 and 12 (Figure 4D), supporting the previously identified inter‐chromosomal interaction preferences observed from the Hi‐C data (Figure S2D, red arrows in the second heatmap). Notably, the preference for inter‐chromosomal interactions between chromosomes 8 and 9 was retained in the trisomic mESCs (Figures 4D and S2D). These results suggest that chromosomes maintain their territories and preferred interacting partners, but reduce their volume in Ts8 mESC compared with WT mESC.

To validate the relationship between chromosomal structures and gene transcription, we combined chromosome painting with nascent RNA imaging in single cells (Section 4; Figure 4A). By quantifying the transcriptional activity of a chromosome as the nascent RNA yield from the chromosome within 1 h, chromosome 9 showed higher transcriptional activity compared with chromosome 8 (Figure 4E), consistent with chromosome‐level expression computed from RNA‐seq data (Figure 2C). Notably, chromosome 8 displayed a reduced level of nascent RNA transcription in Ts8 mESC compared with WT mESC (Figure 4E), concurrent with its compaction in chromosome volume (Figure 4C). Finally, we compared the nascent RNA transcription between intermingling and non‐intermingling regions of chromosomes. Intermingling nuclear regions co‐occupied by two chromosomes were transcribed more actively (Figure 4F), consistent with the Hi‐C results that inter‐chromosomal interactions correlate with active transcription (Figure 3B). Taken together, coupled chromosome painting and nascent RNA imaging validate high transcriptional activity at chromosome intermingling regions, and reveal that trisomic chromosomes have reduced volume and nascent RNA transcription than their normal counterpart.

### 2.5 Transcriptional upregulation of trisomic chromosomes

**FIGURE 5:**
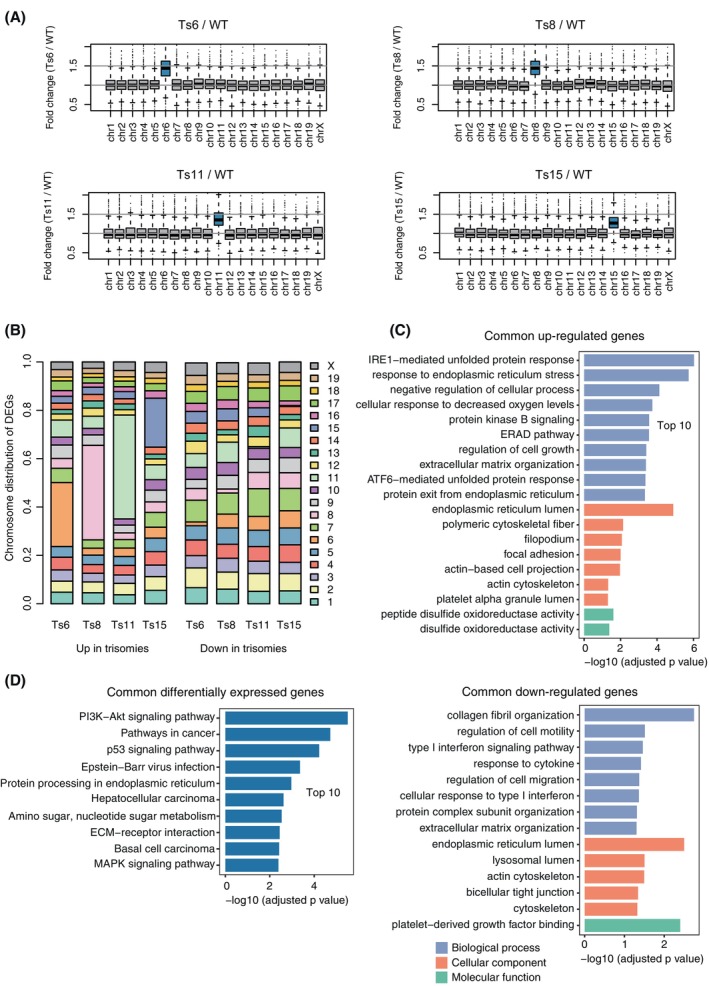
Transcriptional upregulation of trisomic chromosomes. (A) The distribution of expression fold changes between trisomic and WT mESCs of the genes on each chromosome. Genes whose FPKMs less than 1 in both comparing samples are not included. (B) The distribution of differentially expressed genes (DEGs) on different chromosomes. (C). Gene ontology (GO) enrichment analysis of shared up‐ and downregulated genes in trisomic relative to WT mESCs. (D) KEGG pathway enrichment analysis of shared differentially expressed genes in trisomic relative to WT mESCs. KEGG, Kyoto Encyclopedia of Genes and Genomes; mESC, mouse embryonic stem cell; WT, wild‐type.

We further explored the RNA‐seq data of WT and trisomic mESCs. Principal component analysis (PCA) of gene expression values of all genes or excluding the genes on the four trisomic chromosomes (genes on chromosomes 6, 8, 11 and 15) showed high reproducibility between replicate samples, indicating that an extra copy of a chromosome induces distinct global gene expression changes in trisomic mESCs (Figure S5A). Genes on trisomic chromosomes were overall upregulated in trisomic mESCs compared with WT mESC, while genes on the disomic chromosomes (two copies) showed similar expression levels between WT and trisomic mESCs (Figure 5A). Specifically, between 1175 (Ts15) and 1590 (Ts11) genes were upregulated (fold change >1.2, adjusted *p* value < 0.05), with a large proportion of these genes located on the respective trisomic chromosomes (Figures 5B and S5B; Supplementary File 2). Interestingly, the average upregulation of the genes on the trisomic chromosomes was slightly less than the expected 1.5‐fold (three copies vs. two copies) (Figure 5A), suggesting moderate dosage compensation of the genes on the trisomic chromosomes in mESCs.^68^, ^69^ However, studies using other cell lines and tissues have shown an expected expression increase of 1.5‐fold for trisomic chromosomes.^70^, ^71^ Reconciling these differences needs further studies in more cell types and aneuploidy context.

We then investigated whether there are commonly dysregulated genes across the trisomic mESCs. We identified more than 900 genes that were upregulated in at least two of the four trisomic mESCs compared with WT mESC (1.2‐fold, adjusted *p* value < 0.05) and more than 900 shared downregulated genes (Figure S5C; Supplementary File 3). This filtering method depletes upregulated genes specific to a particular trisomic chromosome and enriches globally dysregulated genes shared across different trisomic mESCs. The shared upregulated genes were enriched with gene ontology (GO) terms such as ‘unfolded protein response’, ‘response to endoplasmic reticulum stress’ and ‘response to decreased oxygen levels’ (Figure 5C; Supplementary File 4), consistent with the stresses and energy costs of extra translation, protein folding and protein degradation in trisomic mESCs.^72^ Kyoto Encyclopedia of Genes and Genomes (KEGG) pathway analysis showed that the shared dysregulated genes were enriched with cancer‐related pathways (Figure 5D), consistent with enhanced teratoma formation capacity of these trisomic mESCs.^44^ These results confirm that trisomic chromosomes impact the transcriptome mainly through dosage effects,^73^ but elicit global transcriptome changes that contribute to common cellular phenotypes of aneuploid cells.^18^

## 3 DISCUSSION

Aneuploidy frequently occurs in both cancer and developmental disorders like Down syndrome, where its functional consequences implicated in dosage effects on gene expression and global perturbation of stress response and cell proliferation pathways.^7^, ^74^ However, how aneuploidy affects spatial genome organization remains less understood. Previous imaging studies of chromosome territories lacked gene‐level resolution,^34^, ^75^, ^76^ while Hi‐C studies of cancer cells measure 3D genome alterations due to not only aneuploidy but also other types of genomic aberrations.^37^, ^42^ In this study, we utilized isogenic WT and trisomic mESCs to minimize the confounding effects arising from different genetic backgrounds and diverse types of mutations observed in cancer cells. This approach allows us to better detect the specific effects of trisomy on spatial genome organization.^43^, ^65^

**FIGURE 6:**
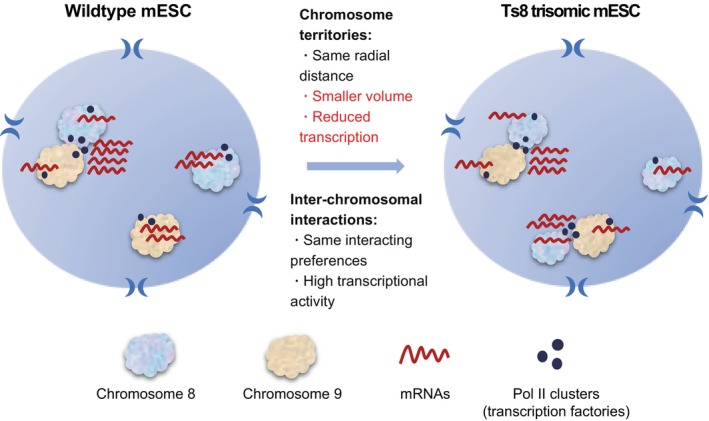
Chromosome territories, inter‐chromosomal interactions and gene transcription in WT and trisomic mESCs. Hi‐C, RNA‐seq and imaging data showed reduced chromosome volume of both trisomic and disomic chromosomes. Radial location of chromosomes and inter‐chromosomal interaction preferences were retained in trisomic mESCs, and chromosome intermingling regions were associated with active transcription in both WT and trisomic mESCs. mESC, mouse embryonic stem cell; WT, wild‐type.

Consistent with previous studies,^73^ we observed the transcriptional upregulation of trisomic chromosomes. This primary dosage effect due to trisomy may cause secondary transcriptional changes through trans‐acting factors and proteomic stress response.^77^, ^78^ This observation offers an explanation for the shared dysregulated pathways and phenotypes across different trisomic cells.^18^, ^44^ Previous imaging studies of trisomic cells reported chromosome compaction in either trisomic chromosomes^34^ or disomic chromosomes.^65^ Using coupled chromosome painting and nascent RNA imaging in single cells, we observed contracted chromosome volume concomitant with weakened transcriptional activity for chromosome 8 in Ts8 mESC compared with WT mESC, but no significant radial relocation of chromosome territories in Ts8 mESC (Figure 6). Further studies are warranted to demonstrate whether such chromosome compaction is a characteristic feature of other aneuploid chromosomes or cell types, and how they are related to the moderate dosage compensation observed in trisomic chromosomes.

Using Hi‐C experiments, we investigated the effect of trisomy on chromatin structures and interactions. Our findings indicate that trisomy does not alter spatial genome structures at the TAD boundary, TAD strength and A/B compartment level. Inter‐chromosomal interaction preferences, as derived from Hi‐C data, remain similar between WT and trisomic mESCs. Importantly, we identified a correlation between inter‐chromosomal interactions and chromatin regions characterized by high gene density, active histone modifications and high transcription levels. This observation is consistent with the recent finding that chromosome intermingling regions and inter‐chromosomal interaction hubs are associated with active transcription.^48^, ^57^ Our findings from coupled chromosome painting and nascent RNA imaging, along with other studies, support that chromosome intermingling regions are associated with higher transcription activities.^79^ Notably, transcription factories have been implicated in promoting inter‐chromosomal interactions through co‐transcription of multiple chromosomal regions.^80^, ^81^ The advancement of multi‐modal imaging techniques and multi‐way interaction sequencing holds promise for elucidating the spatial relationships of various nuclear bodies with respect to chromosome territories and their roles in inter‐chromosomal interactions and RNA biogenesis.^48^, ^82^, ^83^

In summary, we have studied the effect of trisomy on spatial genome organization using an integrated sequencing and imaging approach. Our multi‐modal approach and data resources will facilitate further investigations into the functions and disease relevance of aneuploidy.

## 4 MATERIALS AND METHODS

### 4.1 Cell cultures

WT and trisomic mESCs were cultured on a layer of γ‐irradiated mouse embryonic fibroblasts (i‐MEFs) in KnockOut DMEM (Gibco, 10829018) supplemented with 15% fetal bovine serum (FBS; Sigma, F4135), 1000 U/mL leukaemia inhibitory factor (LIF; ESG1107), 2 mM GlutaMAX‐1 (Gibco, 35050061), 1% non‐essential amino acids (NEAA) (Gibco, 1140050) and 100 μM β‐mercaptoethanol (Gibco, 21985023). Cells were cultured at 37°C in a 5% CO_2_ incubator, and were dissociated into single‐cell suspension with 0.05% Trypsin–EDTA (Gibco, 25300062) at 37°C for 10 min. For the following RNA‐seq and Hi‐C experiments, cells were then sedimented in the cell culture dishes for 30 min to remove the feeder cells.

### 4.2 RNA‐seq experiments

Total RNA of WT and trisomic mESCs was isolated following the TRIzol RNA extraction method, and poly(A) based mRNA enrichment was performed. Then mRNA was fragmented and cDNA was synthesized from mRNA library. RNA‐seq library was prepared following DNA end repair, dATP tailing, adapter ligation and PCR.

### 4.3 Hi‐C experiments

Hi‐C experiment was performed following the in situ Hi‐C protocol.^28^ Briefly, mESCs were digested and crosslinked in 1% formaldehyde solution for 10 min at room temperature, and the reaction was quenched in 0.2 M glycine solution for 5 min. Cell pellet was washed with phosphate‐buffered saline. Crosslinked cells were lysed in ice‐cold Hi‐C lysis buffer and treated with SDS. SDS was then quenched with Triton X‐100. Chromatin was digested with *Mbo*I restriction enzyme and digested overhang was labelled with biotin‐14‐dATP. DNA fragments were ligated. After that, crosslinking was reversed and DNA was purified. DNA was sheared to 300–500 bp and biotin‐labelled DNA was enriched using Dynabeads MyOne Streptavidin T1 beads. The following library construction steps were performed on beads. Briefly, sheared DNA ends were repaired and ligated with dATP tailing. Then Illumina adapters were ligated to DNA fragments and 8–10 cycles of PCR was performed. Finally, PCR products were purified and size‐selected using AMPure beads for Illumina sequencing.

### 4.4 RNA‐seq data analysis

All RNA‐seq reads were aligned to mouse mm10 reference genome using Hisat2^84^ with default parameters and assigned to each gene using the summarizeOverlaps function in Bioconductor.^85^ Low FPKM values were usually experimental or sequencing noise rather than truly expressed genes, so genes whose FPKM values less than 1 in both comparing samples were removed and not included in the downstream analysis.^86^ DESeq2^87^ was used to identify differentially expressed genes (FDR <0.05, fold change >1.2). Differentially expressed genes in at least two trisomic mESCs compared with WT mESC were regarded as shared differentially expressed genes. Enrichr was used to perform GO and KEGG pathway enrichment analysis.^88^

### 4.5 Hi‐C data pre‐processing

Hi‐C pre‐processing was performed using the HiC‐Pro pipeline.^89^ Briefly, sequencing reads were aligned to the mouse mm10 reference genome using the two‐step mode. Unaligned read pairs, singletons and multiple aligned read pairs were discarded. Uniquely mapped read pairs were mapped to *Mbo*I restriction fragments. Then, the invalid read pairs and PCR duplicates were removed. Valid read pairs were used to build Hi‐C contact maps. For the following steps, Hi‐C raw matrices were used for inter‐chromosomal interaction analysis, and ICE^45^ normalized matrices were used for intra‐chromosomal interaction analysis, such as A/B compartment and TAD calling.

### 4.6 Inter‐chromosomal proximity score

Inter‐chromosomal proximity scores were computed as the log_2_ ratio between the observed and expected pairwise inter‐chromosomal interactions. The calculation of expected inter‐chromosomal interactions was modified following Lieberman‐Aiden et al.^25^ First, we calculated the expected proportion of pairwise inter‐chromosomal interactions R_i,j_ = (*N*_i_/*N*)*(*N*_j_/*N*), where *N*_i_ is the total number of observed inter‐chromosomal interactions involving chromosome i, and *N* is the total number of all inter‐chromosomal interactions. This calculation generated an n*n matrix of expected proportions of pairwise inter‐chromosomal interactions, whose diagonal elements are all 0 and the matrix sum R_sum_ is less than 1 (*n* is the number of chromosomes). Then, the expected proportion matrix R was normalized to make the matrix sum equal to the sum of the observed inter‐chromosomal interaction matrix and get the expected inter‐chromosomal interaction matrix E_i,j_ = R_i,j_/R_sum_*O_sum_, where R_sum_ is the sum of expected proportion matrix, and O_sum_ is the sum of the observed inter‐chromosomal interaction matrix. Then, the inter‐chromosomal proximity scores P_i,j_ were computed as the log_2_ ratio between the observed and expected pairwise inter‐chromosomal interactions, P_i,j_ = log_*2*_(O_i,j_/E_i,j_), where O_i,j_ is the observed inter‐chromosomal interactions between chromosome i and chromosome j.

### 4.7 CNV detection from Hi‐C data

CNVkit^90^ was used to detect CNV from Hi‐C data. Using WT mESC data as reference, the whole‐genome sequencing mode was used to detect copy number changes of each trisomic sample relative to WT.

### 4.8 A/B compartment analysis

A/B compartments were detected using R package HiTC.^91^ Briefly, principal component analysis was performed on the correlation matrices generated from the observed/expected interaction matrices (ICE‐normalized, resolution: 500 kb). The average gene density was calculated for the regions with negative and positive principal component 1, respectively. The regions with higher and lower gene density were assigned as compartments A and B, respectively. For the calculation of A/B compartment segregation score, contacts within 2 Mb were removed since they usually correspond to interactions within TADs.^92^

### 4.9 TAD analysis

TADs were identified using Perl script matrix2insulation.pl.^47^ Briefly, ICE‐normalized 40‐kb interaction matrices were used to calculate insulation scores for each 40‐kb bin. The local minimums of insulation scores were regarded as TAD boundaries, and regions between TAD boundaries were regarded as TADs. TADs smaller than 200 kb were removed for subsequent analysis.

### 4.10 ChIP‐seq data analysis

Sequencing reads were aligned to the mouse mm10 reference genome. Read pairs with mapping quality lower than 10 and PCR duplicates were discarded. MACS2 was used to call signal peaks.^93^

### 4.11 Nascent RNA labelling and chromosome painting

The nascent RNA was labelled with metabolic transcription by directly adding uridine analogues 5‐Ethynyluridine (5‐EU, Abcam #ab146642) for a time pulse.^94^, ^95^, ^96^ The 5‐EU labelling of nascent RNA enables microscopic analysis of the distribution of labelled RNA and monitors the dynamics of transcription. The 5‐EU is taken up by cells, converted to nucleotide phosphates, and then incorporated into nascent RNAs by all three RNA polymerases. In the subsequent analysis, we analysed the overlapping regions of nascent RNA and DNA, thus excluding the nascent rRNAs in the nucleoli. The 5‐EU was connected to the fluorophore Alexa 647 with the Click reaction (Click‐iT® EU Alexa Fluor® 647 Imaging Kit, ThermoFisher #C10640). When mESCs reached 70%–80% confluence on the coverslips, Alexa 647‐5‐EU was added to the culture medium at the concentration of 1 mM for 1 h. After that, cells were fixed with a mixture of methanol and acetic acid (3:1) under −20°C for 20 min.^66^ Then the samples were labelled with commercial multicolour FISH probes for specific mouse chromosomes (XMP 8/9/12, MetaSystems) and proceeded following established FISH procedures.^66^

### 4.12 Image acquisition

The 3D stack images were taken on a high‐resolution spin‐disk confocal microscope (Dragonfly), with 0.4 μm step size by 100× oil immersion objective. For each cell line, we selected approximately 30 fields of view (FOVs), with the number of cells between 1 and 10 cells per FOV. The signals of the nascent RNA (Alexa 647‐5‐EU) were excited with a 647 nm laser, the signals of chromosome 9 (XMP 9 Orange) and chromosome 12 (XMP 12 Orange) were acquired with 561 nm channel, the signals of chromosome 8 (XMP 8 Green) were acquired with 488 nm channel, and the signals of nucleus (ProLong® Diamond Antifade Mountant with DAPI) were acquired with 405 nm channel.

### 4.13 Image data analysis

The reconstructed 3D rendering from raw images was implemented in Imaris (9_2_0). According to the spatial distribution of signal intensity, the signals of nucleus and chromosomes were masked and quantified with item cell and nucleus, respectively, and the signals of nascent RNA spots were masked and counted with item vesicles. In this way, quantitative information such as their 3D morphology, location, quantity and intensity can be obtained. Then, we exported the spatial statistics of these features for down‐stream statistical analysis. To remove the variation due to the size of cells, we normalized the feature statistics relative to the diameter/volume of the nucleus.

## AUTHOR CONTRIBUTIONS

C.L., Y.S. and Y.H. designed and supervised the study. M.L. and R.X. performed sequencing experiments and data analysis. J.Y. performed imaging experiments and data analysis. M.Z. generated the mESC cell lines and prepared the biological samples. Y.L., J.H., T.L. and P.W. participated in data analysis and interpretation. M.L., J.Y., M.Z. and C.L. wrote the manuscript. All authors revised and approved the manuscript.

## CONFLICT OF INTEREST STATEMENT

The authors declare no conflicts of interest.

## Supporting information


**Figure S1.** Quality and reproducibility of Hi‐C data. (A) The relationship between contact probability and genomic distance in all the 14 replicate samples (3 replicates each for WT, Ts6, Ts8, Ts11 mESCs and 2 replicates for Ts15 mESC). (B) Pearson's correlation coefficient matrix of genome‐wide raw interaction matrices (bin resolution: 1 Mb). (C) Scatter plot of genome‐wide raw interaction matrices for WT mESC and E14 cell line (bin resolution: 1 Mb). Pearson's correlation coefficients are displayed in the figure. (D) Representative Hi‐C raw interaction matrices of WT mESC and E14 cell line (bin resolution: 100 kb, sequencing‐depth normalized). (E) Hi‐C interaction matrices of chromosome 8 in WT, Ts6, Ts8, Ts11 and Ts15 mESCs (bin resolution: 100 kb, ICE‐normalized) and differential interaction matrices between sample pairs.

**Figure S2.** Copy number effect and inter‐chromosomal interactions from Hi‐C data. (A) Genome‐wide Hi‐C raw interaction matrices of WT and trisomic mESCs (bin resolution: 1 Mb, sequencing‐depth normalized). Below the heatmaps are column sums of corresponding genomic bins. Trisomic chromosomes have increased interactions with other chromosomes due to the extra copy of a chromosome. (B) Chromosome copy numbers in the trisomic mESCs relative to WT mESC, inferred from Hi‐C sequencing data. (C) The total number of inter‐chromosomal interactions for each chromosome in WT and trisomic cells (sequencing‐depth normalized). As indicated by the arrowheads, trisomy results in relatively increased inter‐chromosomal interactions of trisomic chromosomes. (D) Inter‐chromosomal proximity scores between each chromosome (rows) and chromosomes 6, 8, 11 and 15 (individual heatmaps from left to right) in WT and trisomic mESCs (columns).

**Figure S3.** Clustering chromosomes by inter‐chromosomal interaction scores. The inter‐chromosomal proximity scores between all pairs of chromosomes are shown in a heatmap and clustered based on row‐wise and column‐wise similarities. The chromosomal gene density and chromosome‐wide gene expression are displayed on the top of the heatmap.

**Figure S4.** Chromosome radial distance as measured by chromosome painting. The distribution of the distance between the surface of chromosome to the closet nucleus membrane for chromosomes 8, 9 and 12 in WT and trisomic mESCs. Two‐sided Wilcoxon rank‐sum test. From left to right: *s* value = 0.2177, 0.4279 and 0.2876; *n* = 361, 306, 441, 215, 368 and 110 chromosomes. NS: not significant.

**Figure S5.** Quality control of gene expression data.
(A) The PCA results of RNA‐seq replicates using all genes (left) or excluding genes on all the trisomic chromosomes 6, 8, 11 and 15 (right). Dots with the same colour represent the three replicates for each sample type. (B). The percentage of differentially expressed genes per chromosome (chromosomes 1–19 and X) in trisomic mESCs. (C) Venn diagrams of the number of shared up‐ and downregulated genes among different trisomic mESCs relative WT mESCs.

**Supplementary File 1.** Hi‐C reads filtering statistics.

**Supplementary File 2.** Differentially expressed genes in trisomies.

**Supplementary File 3.** Shared differentially expressed genes.

**Supplementary File 4.** Enriched GO terms and KEGG pathways.

**Table S1.** Public data sets used in this paper.

## Data Availability

All Hi‐C and RNA‐seq data generated in this study have been deposited in the GEO database under the accession number GSE179435. The public data sets used in this study are listed in Table S1.
