# Supplementary figures and images for: The effect of trisomic chromosomes on spatial genome organization and global transcription in embryonic stem cells
Source: Cell Prolif. 2024 Mar 29;57(8):e13639. doi: 10.1111/cpr.13639 (PMC11294443; doi:10.1111/cpr.13639)

**Figure S1. Quality and reproducibility of Hi-C data**

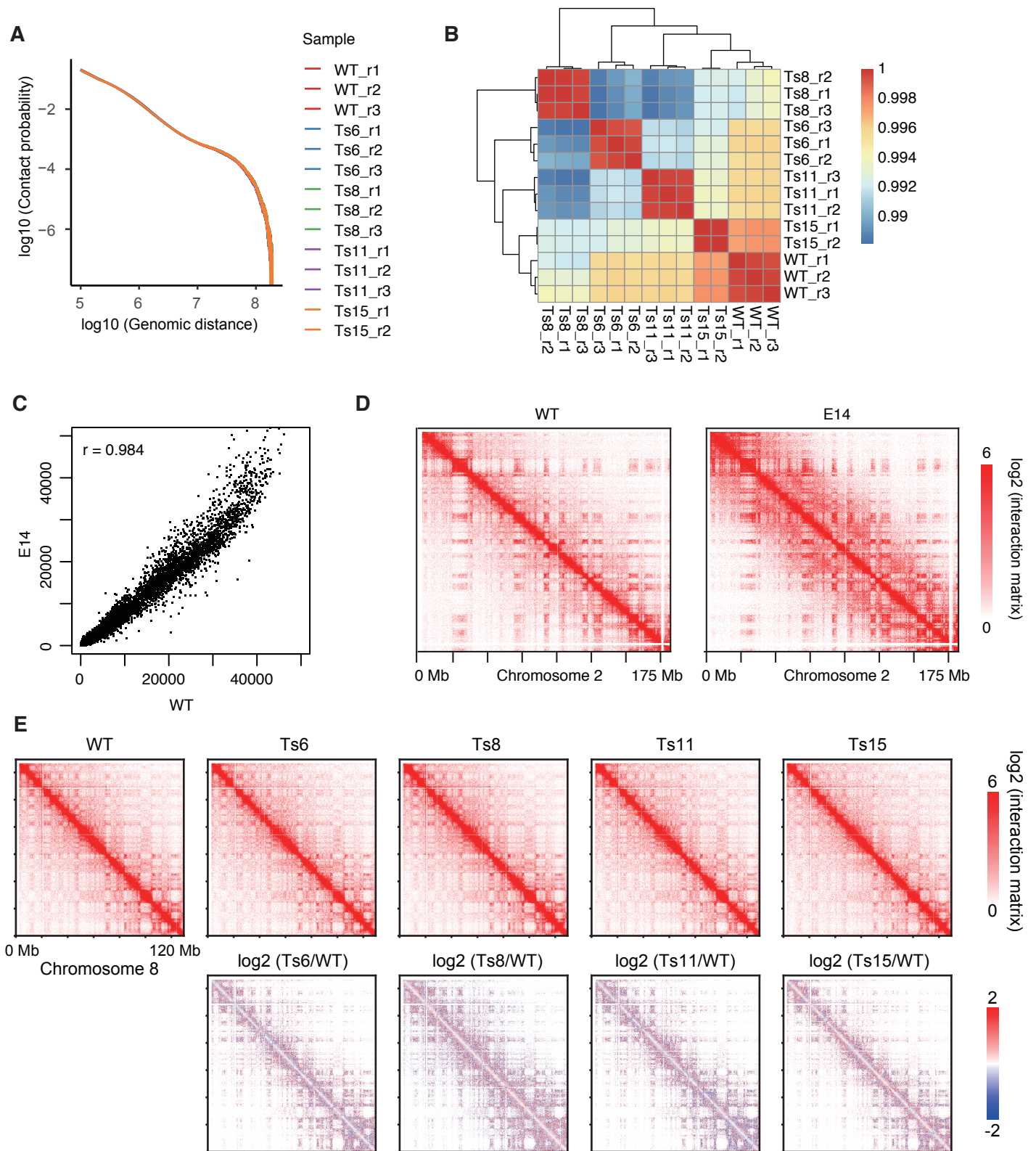

Supplement: Supplementary file 1 — Figure S1. Quality and reproducibility of Hi‐C data. (A) The relationship between contact probability and genomic distance in all the 14 replicate samples (3 replicates each for WT, Ts6, Ts8, Ts11 mESCs and 2 replicates for Ts15 mESC). (B) Pearson's correlation coefficient matrix of genome‐wide raw interaction matrices (bin resolution: 1 Mb). (C) Scatter plot of genome‐wide raw interaction matrices for WT mESC and E14 cell line (bin resolution: 1 Mb). Pearson's correlation coefficients are displayed in the figure. (D) Representative Hi‐C raw interaction matrices of WT mESC and E14 cell line (bin resolution: 100 kb, sequencing‐depth normalized). (E) Hi‐C interaction matrices of chromosome 8 in WT, Ts6, Ts8, Ts11 and Ts15 mESCs (bin resolution: 100 kb, ICE‐normalized) and differential interaction matrices between sample pairs. [file CPR-57-e13639-s007.pdf]

**Figure S2. Copy number effect and inter-chromosomal interactions from Hi-C data**

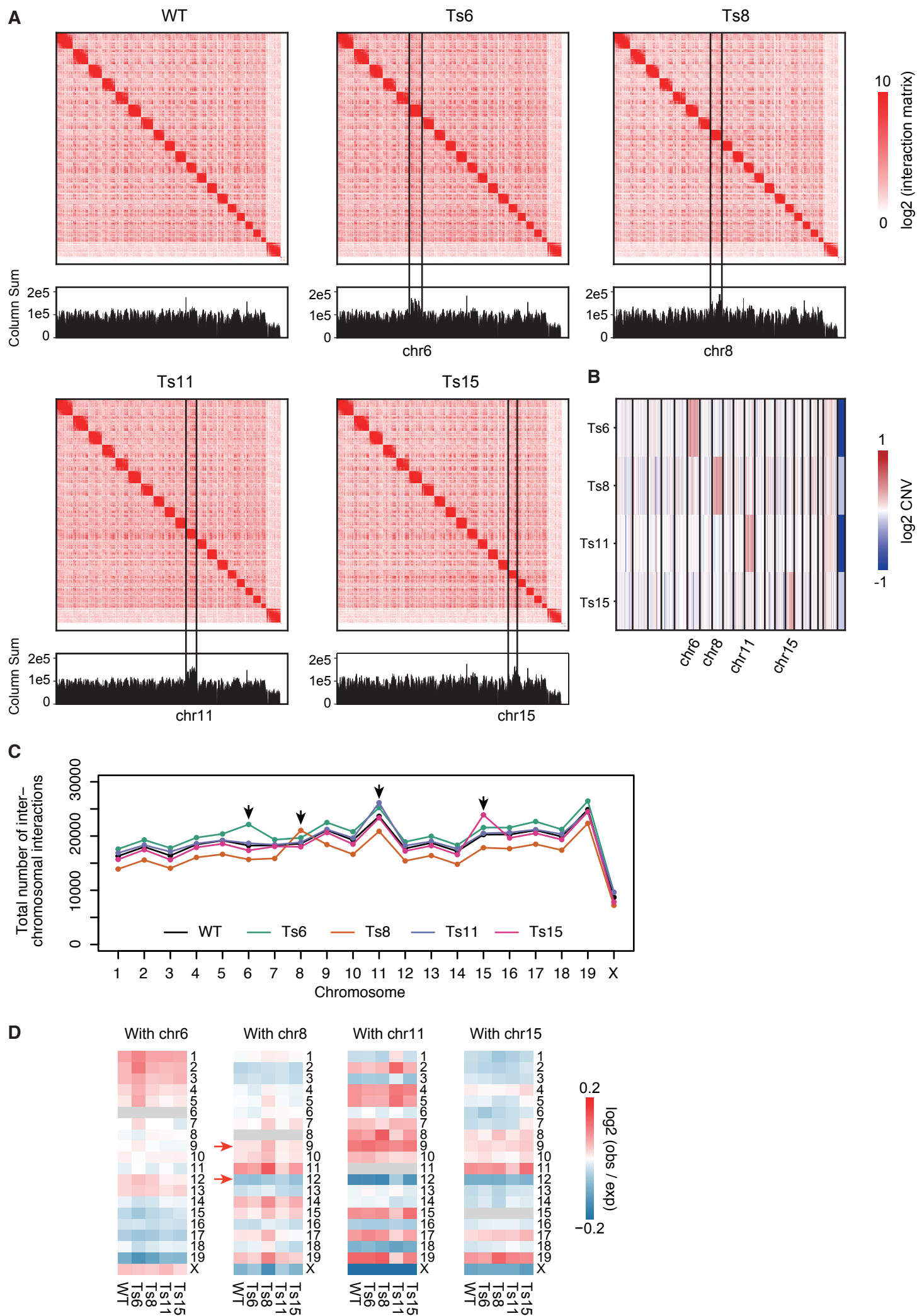

Supplement: Supplementary file 2 — Figure S2. Copy number effect and inter‐chromosomal interactions from Hi‐C data. (A) Genome‐wide Hi‐C raw interaction matrices of WT and trisomic mESCs (bin resolution: 1 Mb, sequencing‐depth normalized). Below the heatmaps are column sums of corresponding genomic bins. Trisomic chromosomes have increased interactions with other chromosomes due to the extra copy of a chromosome. (B) Chromosome copy numbers in the trisomic mESCs relative to WT mESC, inferred from Hi‐C sequencing data. (C) The total number of inter‐chromosomal interactions for each chromosome in WT and trisomic cells (sequencing‐depth normalized). As indicated by the arrowheads, trisomy results in relatively increased inter‐chromosomal interactions of trisomic chromosomes. (D) Inter‐chromosomal proximity scores between each chromosome (rows) and chromosomes 6, 8, 11 and 15 (individual heatmaps from left to right) in WT and trisomic mESCs (columns). [file CPR-57-e13639-s006.pdf]

Figure S3. Clustering chromosomes by inter-chromosomal interaction scores

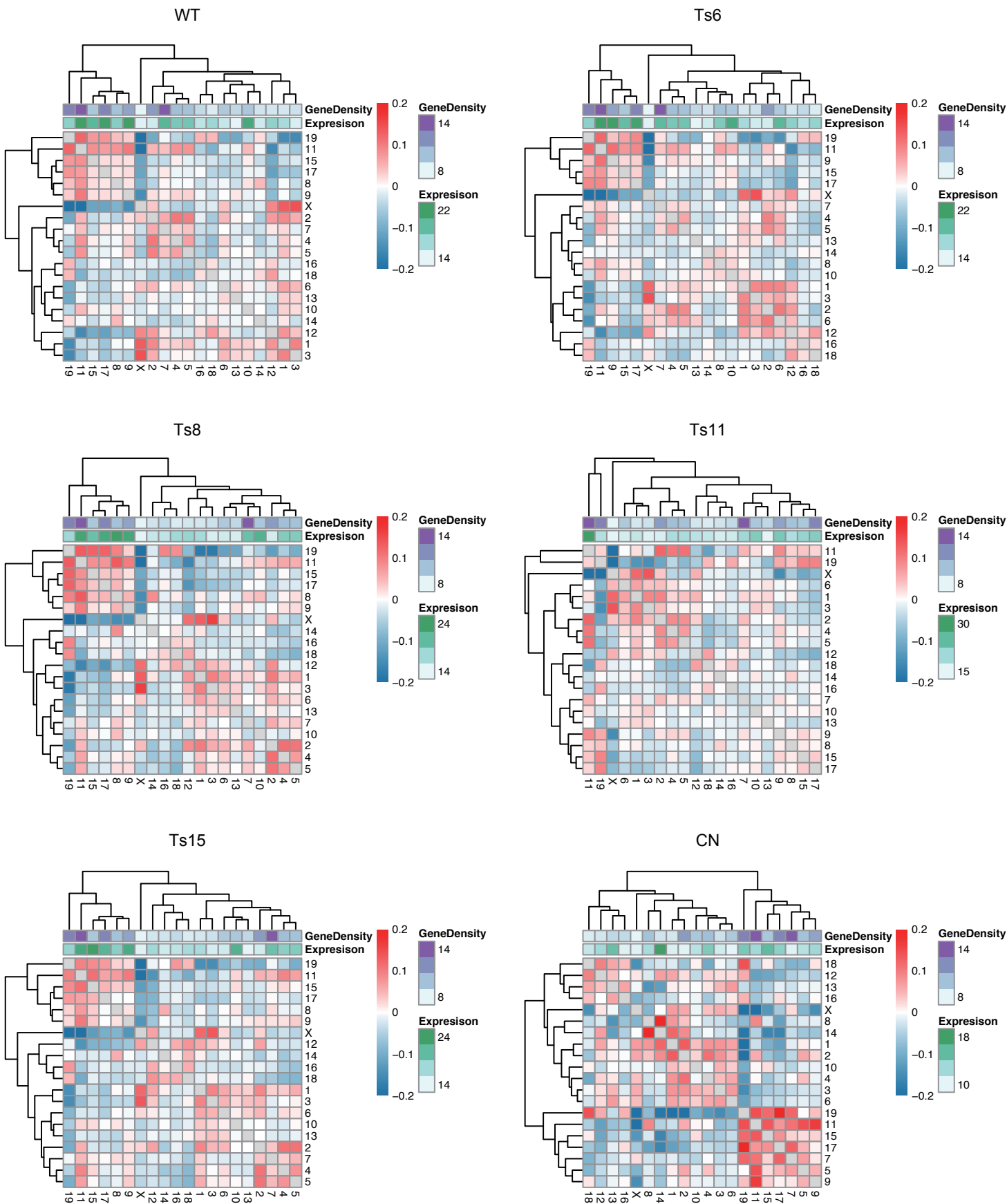

Supplement: Supplementary file 3 — Figure S3. Clustering chromosomes by inter‐chromosomal interaction scores. The inter‐chromosomal proximity scores between all pairs of chromosomes are shown in a heatmap and clustered based on row‐wise and column‐wise similarities. The chromosomal gene density and chromosome‐wide gene expression are displayed on the top of the heatmap. [file CPR-57-e13639-s002.pdf]

**Figure S4. Chromosome radial distance as measured by chromosome painting**

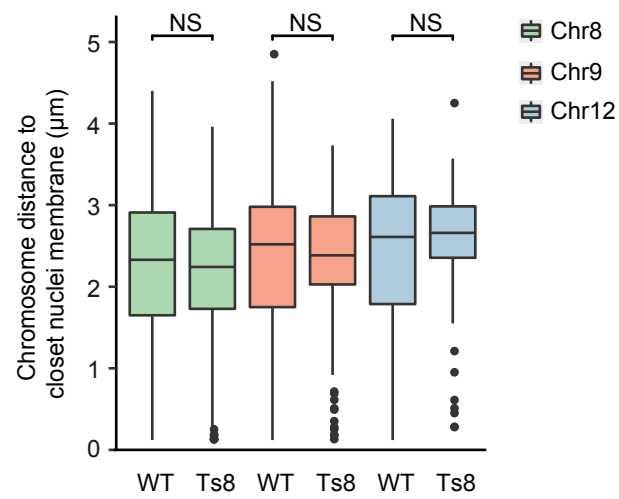

Supplement: Supplementary file 4 — Figure S4. Chromosome radial distance as measured by chromosome painting. The distribution of the distance between the surface of chromosome to the closet nucleus membrane for chromosomes 8, 9 and 12 in WT and trisomic mESCs. Two‐sided Wilcoxon rank‐sum test. From left to right: s value = 0.2177, 0.4279 and 0.2876; n = 361, 306, 441, 215, 368 and 110 chromosomes. NS: not significant. [file CPR-57-e13639-s005.pdf]

**Figure S5. Quality control of gene expression data**

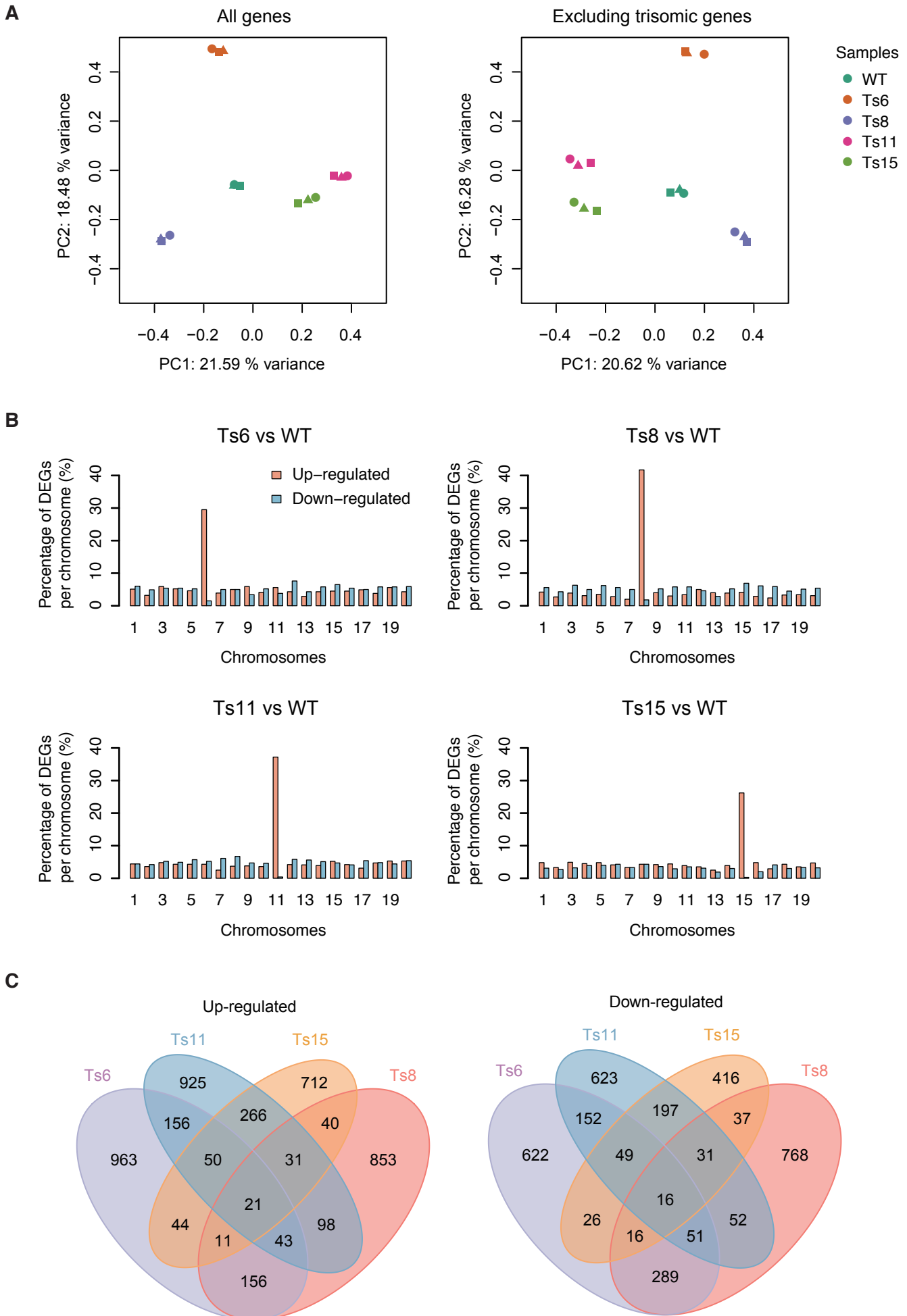

Supplement: Supplementary file 5 — Figure S5. Quality control of gene expression data. (A) The PCA results of RNA‐seq replicates using all genes (left) or excluding genes on all the trisomic chromosomes 6, 8, 11 and 15 (right). Dots with the same colour represent the three replicates for each sample type. (B). The percentage of differentially expressed genes per chromosome (chromosomes 1–19 and X) in trisomic mESCs. (C) Venn diagrams of the number of shared up‐ and downregulated genes among different trisomic mESCs relative WT mESCs. [file CPR-57-e13639-s009.pdf]
