# Supplementary material for: The effect of trisomic chromosomes on spatial genome organization and global transcription in embryonic stem cells
Source: Cell Prolif. 2024 Mar 29;57(8):e13639. doi: 10.1111/cpr.13639 (PMC11294443; doi:10.1111/cpr.13639)
Supplement: Supplementary file 10 — Table S1. Public data sets used in this paper. [file CPR-57-e13639-s010.docx]

Table S1. Public datasets used in this paper.

| Data | Source | Identifier |
| --- | --- | --- |
| E14 H3K4me1 ChIP-seq | ENCODE | ENCSR000CGN |
| E14 H3K4me3 ChIP-seq | ENCODE | ENCSR000CGO |
| E14 H3K27ac ChIP-seq | ENCODE | ENCSR000CGQ |
| E14 Control ChIP-seq | ENCODE | ENCSR095IPH |
| E14TG2a.4 H3K9me3 ChIP-seq | ENCODE | ENCSR857MYS |
| E14TG2a.4 H3K27me3 ChIP-seq | ENCODE | ENCSR059MBO |
| E14TG2a.4 control ChIP-seq | ENCODE | ENCSR326ULS |
| E14 RNA pol II ChIP-seq | Handoko et al., 2011 | GEO: GSE28247 |
| E14 CTCF ChIP-seq | Bonev et al., 2017 | GEO: GSE96107 |
| E14 Hi-C | Bonev et al., 2017 | GEO: GSE96107 |
| NPC Hi-C | Bonev et al., 2017 | GEO: GSE96107 |
| CN Hi-C | Bonev et al., 2017 | GEO: GSE96107 |
| E14 ChromHMM | Pintacuda et al., 2017 | https://github.com/guifengwei/ChromHMM_mESC_mm10 |
